# Deciphering the anti-filarial potential of bioactive compounds from *Ocimum sanctum*: a combined experimental and computational study

**DOI:** 10.1080/13880209.2022.2132030

**Published:** 2022-11-22

**Authors:** Ayushi Mishra, Vipin Kumar, Anchal Singh

**Affiliations:** Department of Biochemistry, Institute of Science, Banaras Hindu University, Varanasi, India

**Keywords:** MD simulation, glutathione *S*-transferase, superoxide dismutase, glutathione reductase, chlorogenic acid, ursolic acid, luteolin

## Abstract

**Context:**

The anthelminthic effect of *Ocimum* species (Lamiaceae) has been reported, however, its anti-filarial effect has not been explored to date.

**Objective:**

This study evaluates the effect of *Ocimum sanctum* L. (OS) against lymphatic filarial parasites.

**Material and methods:**

The ethanol extract of OS (EOS) leaves was tested for anti-filarial activity against *Setaria cervi*. Equal size and number (*n* = 10) of adult female *S. cervi* worms were incubated in 125, 250 or 375 μg/mL EOS extract for 6 h at 37 °C. The OS bioactive components were identified by UPLC-ESI-MS/MS and subjected to docking and molecular dynamics (MD) simulation against filarial antioxidant proteins.

**Results:**

The EOS significantly inhibited the motility of adult female *S. cervi* after 6 h of incubation. The motility was found to be reduced by 53.7% in 375 µg/mL and 43.8% in 250 µg/mL EOS after 6 h of treatment. The UPLC-ESI-MS/MS analysis of ethanol extract of *O. sanctum* revealed the presence of 13 bioactive compounds. The docking analysis showed eight OS bioactive compounds to have high binding affinity (> 4.8 kcal/mol) towards antioxidant proteins of filarial parasites. Additionally, MD simulation studies showed significant impact of (RMSD ≤ 10 Å) chlorogenic acid, luteolin and ursolic acid on filarial antioxidant enzymes/proteins. To our knowledge, this is the first report of the anti-filarial activity of *Ocimum sanctum*.

**Discussion and conclusions:**

The effect of EOS and OS bioactive components on human filarial parasites can be further evaluated for the development of new anti-filarial formulations.

## Introduction

Lymphatic filariasis (LF) is a major health concern of tropical and sub-tropical countries. The disease is caused by three nematode worms: *Wuchereria bancrofti*, *Brugia malayi*, and *Brugia timori*. Presently 893 million people in 49 countries are living at the risk of LF (Cromwell et al. 2020). The World Health Organisation (WHO) sponsored the Global Program to Eliminate Lymphatic Filariasis (GPELF) and recommends Triple Drug Therapy to block the transmission of Lymphatic Filariasis. The triple drug therapy comprises drugs ivermectin (IVM), diethylcarbamazine (DEC), and albendazole which have to be administered to the entire population living in endemic areas. These drugs are effective only on the larval stages and are completely ineffective on adult worms (Wadhawan et al. 2014). Several adverse effects are associated with anti-filarial drugs which include fever, headache, myalgia, fatigue, hypertension, vomiting, cough, seizures, vision problems, etc. (Behera and Bhatnagar 2018). Hence, there is an urgent need to find anti-filarial drugs with adulticidal activity and minimal side effects.

Adult filarial worms are long lived and easily survive for a period of 8–10 years inside their human hosts. Filarial worms have efficient antioxidant systems which protect them from reactive oxygen species generated by host macrophages, neutrophils, and eosinophils. The antioxidant enzymes superoxide dismutase (SOD), glutathione S-transferase (GST), glutathione reductase (GR), and thioredoxin reductase (TrxR) are established helminth drug targets and studies confirm that inhibiting one or more of these activities proves fatal for the parasites (Gupta and Rathaur 2005; Gupta and Srivastava 2006; Rathaur et al. 2001; Joardar et al. 2021).

The genus *Ocimum* (Lamiaceae) encompasses most popular medicinal herbs and culinary ingredients. *Ocimum basilicum* L., *Ocimum gratissimum* L., and *Ocimum sanctum* L. are extensively studied for their therapeutic properties. The ethanol extract of *Ocimum basilicum* (OB) is hypocholesterolemic, hypolipidemic, and can modulate the activity of macrophage surface scavenger receptors (Bravo et al. 2008). The OB leaf extract was also shown to minimize drug induced nephrotoxicity in mice (Karaali et al. 2018). The herb *Ocimum gratissimum* (OG) is widely consumed across the world for its medicinal and nutritional value and its alcohol extract can counteract cyclophosphamide induced nephrotoxicity (Alabi et al. 2021) and lead acetate toxicity in Wistar rats (Oyem et al. 2021). *Ocimum sanctum* (OS) is an herbaceous plant, rich in medicinal properties. *Ocimum sanctum* is also known as the elixir of life in Ayurveda because of its curative effects. In India OS is considered a holy plant and is grown in almost every household. *Ocimum species* are found in India, Malaysia, Saudi Arabia and is widely distributed in tropical and sub-tropical countries. The plant is a potent antioxidant and has immunomodulatory, antimicrobial, anticancer, anti-HIV properties (Rege et al. 2010; Mallikarjun et al. 2016). It is widely used for the treatment of cough, headache, cancer, stress, diseases of the CNS, Leishmaniasis, etc. (Pattanayak et al. 2010; Bhalla et al. 2017; Venuprasad et al. 2017).

The aerial parts of OS are rich in several phenolic compounds and terpenoids such as rosmarinic acid, ursolic acid, and eugenol which impart medicinal properties to this plant. The essential oils of *Ocimum basilicum* have antioxidant activity that is comparable to the synthetic antioxidant tertiary butylhydroquinone (Farouk et al. 2016). OS leaf extract was also found to be effective in the reduction of eggs and larvae count in sheep gastrointestinal nematodes (Kanojiya et al. 2015). A previous *in vitro* study (Sousa et al. 2021) on nematode *Haemonchus contortus* showed the anti-helminthic potential of *O. basilicum* essential oils. Although the anthelminthic activities of *Ocimum* species and its essential oils have been studied, nevertheless, the anti-filarial effect of *Ocimum* sp. has not been investigated to date. Considering the traditional use of *Ocimum* sp. as a medicinal herb and its potent anthelminthic activity we wondered if *Ocimum* sp. can have anti-filarial effects too. To test this hypothesis, the effects of OS leaf extract and OS bioactive compounds on a model lymphatic filarial parasite *S. cervi* were evaluated.

## Materials and methods

### Ethics statement

This study does not involve the use of experimental animal or human samples. The *Setaria cervi* worm is found in the peritoneal cavity of Indian water buffaloes which are slaughtered for table purposes in local slaughter houses. The *S. cervi* worms were procured from the peritoneal lavage of freshly slaughtered buffaloes.

### Plant material collection and extract preparation

Leaves of *Ocimum sanctum* were collected between September and November 2020 from the botanical gardens of Banaras Hindu University (between latitude 25.267878 and longitude 82.990494), Varanasi, UP, India. The botanical identification was made by the taxonomist Prof. Shashi Pandey at the Botany Department, Institute of Science, Banaras Hindu University. Leaves were washed, shade-dried, ground to a fine powder in mortar and pestle, and stored at 4 °C for extract preparation. A 10% ethanol extract was prepared from OS leaf powder in a Soxhlet apparatus (Redfern et al. 2014). Later, the ethanol was evaporated using a rotary evaporator and the dry extract was stored at −20 °C. The dried OS leaf extract was weighed and solubilized in DMSO (100 mg/mL concentration) every time before use.

### UPLC-ESI-MS/MS analysis of ethanol leaf extract of Ocimum sanctum

The UPLC-ESI-MS/MS analysis was performed on Acquity Ultra Performance Liquid Chromatograph (UPLC), coupled to a Quadrupole-Time of Flight mass spectrometer (QTOF-MS, Synapt G2 HDMS, Waters Corporation, Manchester, UK), equipped with electrospray ionization (ESI). Chromatographic separation of OS extract was performed by ACQUITY UPLC BEH C_18_ column at 35 °C. The mobile phase consisted of A-phase, methanol and water (5:95) and B-phase, methanol and water (95:5) with 0.1% formic acid. Mass Lynx 4.1 software was used for data acquisition and processing. RIKEN tandem mass spectral database (ReSpect) for phytochemicals software was used for detailed analysis of UPLC-ESI-MS/MS data (Sawada et al. 2012).

### Parasites collection and culture

*Setaria cervi* is a widely used WHO recommended model for lymphatic filariasis (Joardar et al. 2021). Adult live female *S. cervi* parasites were recovered from the peritoneal lavage of Indian water buffaloes that were slaughtered for table purposes. Worms were brought to the laboratory in Krebs’s Ringer bicarbonate buffer (KRB) supplemented with streptomycin, penicillin, glutamine, and 0.5% glucose (KRB maintenance medium). Worms were washed with KRB and incubated in KRB maintenance medium at 37 °C in a water bath for 1 h before subsequent treatments (Singh and Rathaur 2005).

### Exposure of S. cervi worms to EOS

Equal size and number (*n* = 10) of adult female *S. cervi* worms were incubated in supplemented KRB having 125, 250, or 375 μg/mL EOS leaf extract for 6 h at 37 °C in a CO_2_ incubator at 95% humidity. Worms incubated in supplemented KRB medium with 0.4% DMSO served as vehicle control.

### Effect of EOS on the motility of S. cervi worms

The motility assessment of EOS treated adult female *S. cervi* worms was done at hourly intervals up to 6 h by an investigator blinded to the experiment. The movement of treated worms was scored with either positive or negative signs by visual inspection. The recovery of filarial worms was checked at the end of the experiment by transferring the worms to fresh KRB maintenance medium (Sharma et al. 2021).

### Estimation of total reactive oxygen species (ROS)

For ROS generation, NBT assay was performed on control and EOS treated adult female *S. cervi* worms by the method of Choi et al. (2006) with some modifications. Precisely, worms were incubated in 2% NBT solution for 1 h at RT followed by successive washings using PBS and methanol. In the next step, worms were incubated in DMSO to dissolve the formazan crystals and the final absorbance was recorded at 620 nm.

### DNA fragmentation analysis

Extraction of total genomic DNA from *S. cervi* adult female parasites was done by the method of Smith and Rajan (2000), with minor modifications. In brief, worms were homogenized in lysis buffer containing 20 mM Tris buffer (pH 8.0), 50 mM EDTA, 0.5% SDS, 100 mM NaCl, 1% β-mercaptoethanol and 0.1 mg/mL proteinase K followed by incubation at 55 °C for 3 h. Phenol–chloroform-isoamyl alcohol in 25:24:1 ratio was used for DNA extraction followed by centrifugation at 10,000 rpm for 10 min. The supernatant was treated with 3 M sodium acetate and 100% cold ethanol, centrifuged at 10,000 rpm for 10 min and the pellet was washed twice with 70% ethanol and dissolved in 10 mM Tris-EDTA (TE) buffer (pH 8.0). The total genomic DNA was separated on 1.8% Agarose gel containing ethidium bromide and bands were visualized using a GelDoc system (Biorad, Hercules, CA).

### Retrieval of target protein structures and validation

3D structure of *Wuchereria bancrofti* glutathione *S*-transferase, pi class (GST) (5D73, DOI: 10.2210/Pdb5D73/pdb) and thioredoxin (Trx) (4FYUA, 10.2210/pdb4FYU/pdb) were retrieved from Protein Data Bank (Burley et al. 2021; http://www.rcsb.org). 3D structure of *Brugia malayi* glutathione reductase (GR) (PM0077742) was retrieved from Protein Model Database (srv00.recas.ba.infn:it/PMDB). The three-dimensional structure of any filarial superoxide dismutase (SOD) was not available in these databases, hence the template sequence of SOD (Accession no. CTP82144.1) was selected based on maximum score and query coverage for protein structure modelling. LOMETS, a meta server approach to template-based protein structure prediction was used to model the structure of *B. malayi* SOD. Validation of protein models was done by Rampage server (mordred.bioc.cam.ac.uk), and PROCHECK servers (Lovell et al. 2003). The quality assessment and hydrogen bond statistics of GST, Trx, GR and SOD models were also checked by VADAR server (Willard et al. 2003). Prediction of the active site of selected 3D structures of GST, Trx, GR, and SOD was done by Metapocket 2.0 server (Huang 2009).

### Retrieval of ligand structures

Based on UPLC-ESI-MS/MS data, EOS bioactive compounds were selected for docking analysis. The structures of ligands were retrieved from PubChem Compound Database (Kim et al. 2021) in SDF format and converted into PDB format with Biovia Discovery Studio 3.5 (https://discover.3ds.com). Lipinski filter was used to predict drug like behaviour of OS bioactive compounds (Lipinski 2004; Jayaram et al. 2012). Absorption, distribution, metabolism, excretion, and toxicity (ADMET) properties of OS bioactive compounds were predicted by admetSAR software (Cheng et al. 2012).

### Molecular docking

The PDB format of all the ligands and target proteins was generated for molecular docking. For docking studies, all the selected OS bioactive compounds were docked against filarial target proteins GST, Trx, GR, and SOD. PatchDock server (parameter RMSD value 1.5, protein complex-small ligand) (Schneidman-Duhovny et al. 2005) and YASARA (Yet Another Scientific Artificial Reality Application) (Krieger and Vriend 2014) tools were used for docking of ligands with target molecules. The best docking configuration of OS bioactive compounds and filarial antioxidant proteins/enzymes was visualized with Biovia Discovery studio 3.5 (Kumar et al. 2022).

### Molecular dynamics simulation

The stability of the filarial antioxidant protein models and ligands interaction was examined by molecular dynamic simulation using NAMD (Nanoscale Molecular Dynamics v 2.14) (Phillips et al. 2005). Ligand PDB file was converted into mol2 file by Open Babel chemical format converter. CHARMM-GUI (Jo et al. 2013) input generator and ligand modeller were used to generate NAMD input files and ligands parameterization. All the files required for MD run were created by VMD (Visual Molecular Dynamics) version 1.9.3 (Humphrey et al. 1996). VMD dispdev command was used for protein and ligand complex file formation. Further, the complex was solvated by a TIP3P water box with a 5 Å layer of water in each direction and was neutralized with 0.15 M NaCl. Molecular dynamics simulation was carried out in the Param Shivay supercomputing facility of IIT BHU. MD run was performed at 310 K temperature, 1000 steps of energy minimization, and 50 ns time trajectory under 3D periodic boundary condition. The results were visualized by VMD for calculating the fluctuation in root mean square deviation (RMSD), root mean square fluctuation (RMSF), and Radius of gyration (Rg) during the simulation run.

### Statistical analysis

All *in vitro* experiments were repeated in triplicates. The data are expressed as mean ± SD which was calculated using Graph Pad Prism 8.1 software (GraphPad Software, La Jolla, CA). The statistical significance between control and EOS treated worms was calculated with Student’s *t*-test. *p < 0.05, **p < 0.01 and ***p < 0.001 was considered statistically significant.

## Results

### Assessment of motility of adult *S. cervi* after EOS treatment

**Figure 1.**
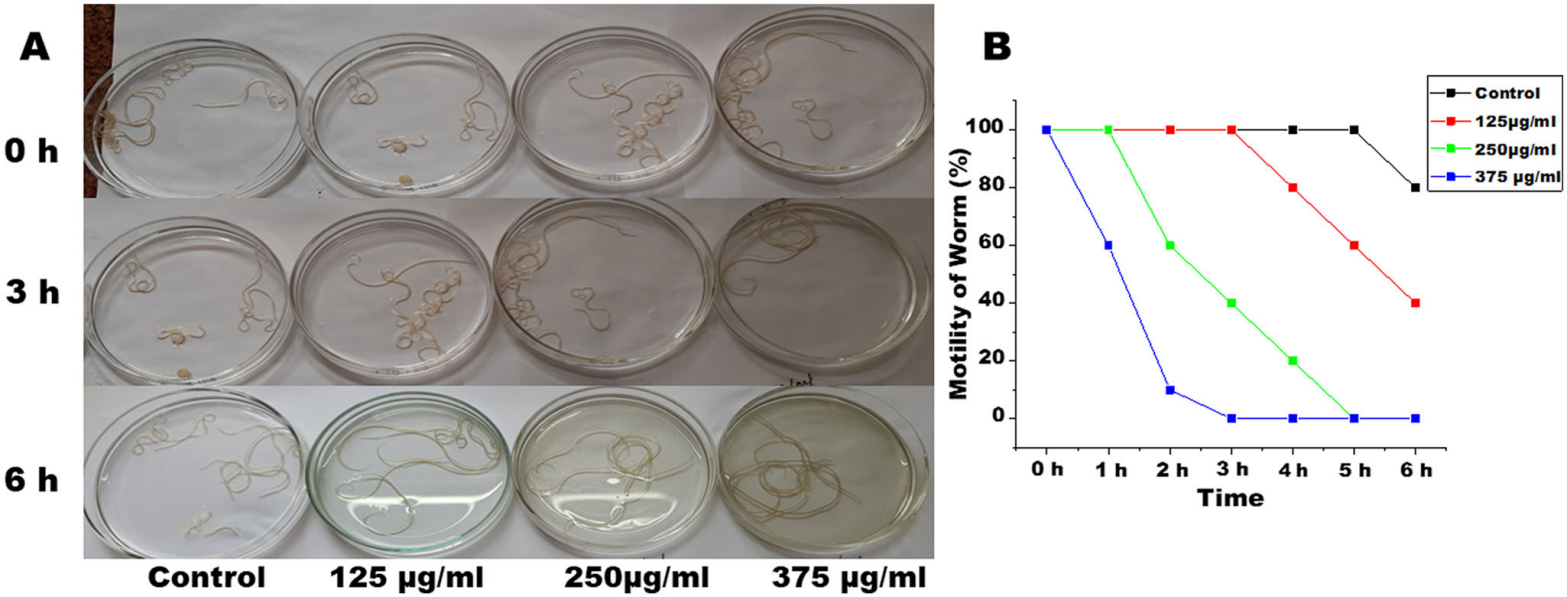
Images of control and EOS treated female *S. cervi* worms (*n* = 10), (A) after 0, 3, and 6 h of treatment. (B) Motility of *S. cervi* worms after treatment was measured in percentage at hourly intervals. Values are mean ± SD of three experiments performed in triplicate.

The adult female *S. cervi* were incubated for 6 h in KRB maintenance medium at 37 °C under 5% CO_2_ and 95% humidity. After completion of incubation, the parasites were transferred to a fresh medium for assessing the recovery. At EOS concentrations of 250 µg/mL and above adult female *S. cervi* became non-motile after 5 h of incubation (Figure 1). The motility reduction of *S. cervi* upon treatment with EOS was found to be time and dose dependent. The recovery of worms after completion of 6 h was checked by transferring female *S. cervi* into fresh KRB maintenance medium for 1 h. The worms treated with 125 µg/mL of EOS were able to revive in a fresh medium, however, parasites treated with concentrations higher than 125 µg/mL did not show any signs of recovery even after completion of the entire recovery period which showed that the effect of EOS is not of temporary nature.

### Effect of EOS extract on total ROS production in S. cervi

**Figure 2.**
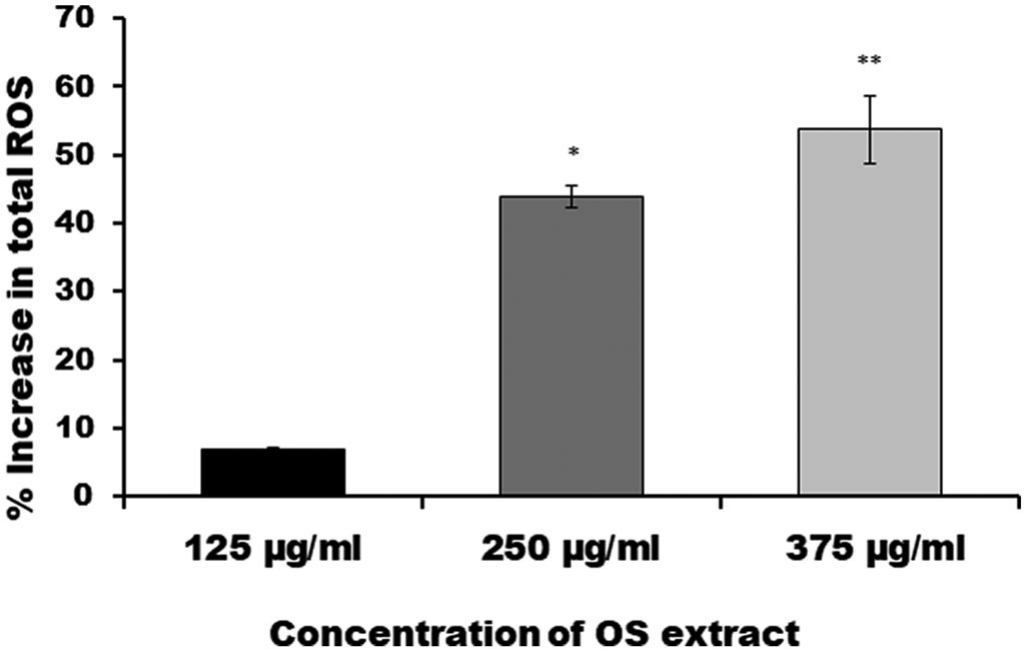
Total reactive oxygen species production assessed in percentage after exposure of adult female *S. cervi* worms with EOS ethanol extract. The data expressed is mean ± SD of three independent experiments. **p < 0.01, *p < 0.05. Values with *p* < 0.05 were considered significant.

The production of reactive oxygen species by adult female *S. cervi* upon exposure to EOS was analyzed by spectrophotometry assay using nitro blue tetrazolium as substrate. Intracellular ROS generation was found to be significantly higher in treated worms as compared to the control parasites (Figure 2). The ROS generation was found to increase by 6.7% in 125 µg/mL, 43.8% in 250 µg/mL (*p*-value ≤ 0.05) and 53.7% in 375 µg/mL (*p*-value ≤ 0.01) EOS-treated worms. The increase in ROS generation upon EOS treatment varied in a concentration-dependent manner.

### Effect of EOS on DNA fragmentation of *S. cervi*

**Figure 3.**
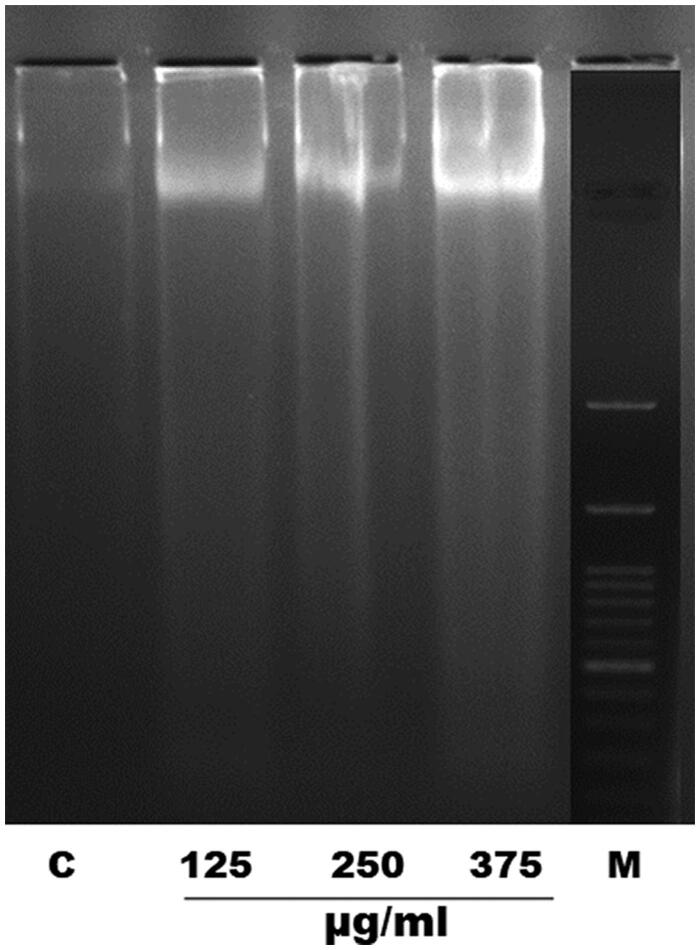
EOS induced DNA fragmentation in adult female *S. cervi* after 6 h of treatment followed by DNA isolation. The isolated DNA from control and treated worms was run on 1.8% agarose gel. C: control, treated parasites (125, 250, 375 µg/mL concentration of OS ethanol extract) and M: marker (molecular weight 100–3000 bp).

The effect of increased ROS levels on cellular DNA was investigated by DNA fragmentation assay. Adult female *S. cervi* worms were treated with different concentrations of EOS and DNA fragmentation was confirmed by extraction of total genomic DNA content of treated and control parasites. The genomic DNA analysis showed dose dependent nucleosomal DNA degradation after 6 h of treatment with EOS. The maximum DNA laddering was observed at 375 µg/mL and least at 125 µg/mL concentration of EOS (Figure 3).

### UPLC-ESI-MS/MS analysis of ethanol extract of OS leaves

**Figure 4.**
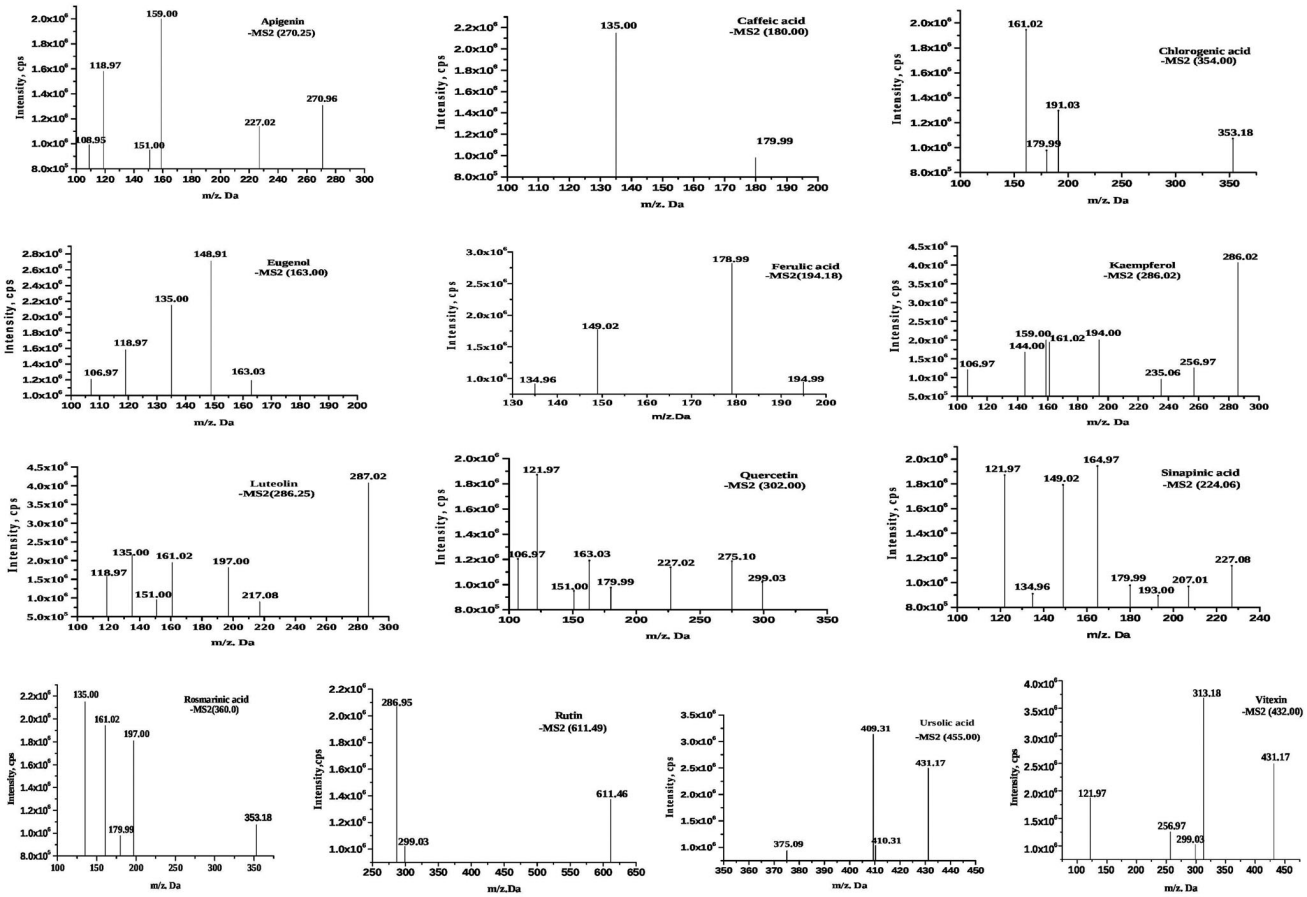
Graphical representation of MS/MS spectra and fragmentation profile of *Ocimum sanctum* bioactive compounds.

The UPLC-ESI-MS/MS analysis was performed for the determination of bioactive compounds from the ethanol extract of *O. sanctum* leaves. The bioactive compounds were identified based on their retardation time and molecular mass with the database ResPect for Phytochemicals (http://spectra.psc.riken.jp/) software. The ethanol fraction contained 13 metabolites, i.e., apigenin, caffeic acid, chlorogenic acid, eugenol, ferulic acid, kaempferol, luteolin, quercetin, rosmarinic acid, rutin, sinapic acid, ursolic acid, and vitexin, which were identified in the positive ion mode (Figure 4). The respective names, formulas, and retention times of all the compounds identified by UPLC-ESI-MS/MS in this study are listed in Table 1.

**Table 1.** Bioactive compounds identified in ethanolic extract of *Ocimum sanctum* by UPLC-ESI-MS/MS.

S. no.	Compound	RT (min)	Formula	(M + H) ^+^	Fragmentation profile (*m*/*z*)
1.	Apigenin	4.87	C_15_H_10_O_5_	270.25	108.95 (9.906e5)
					118.97 (1.580e6)
					151.00 (9.503e5)
					159.00 (1.998e6)
					227.02 (1.137e6)
					270.96 (1.308e6)
2	Caffeic acid	7.05	C_9_H_8_O_4_	180.44	135.00 (2.148e6)
					180.93 (1.182e6)
3	Chlorogenic acid	7.80	C_16_H_18_O_9_	354.09	161.02 (1.945e6)
					179.99 (9.772e5)
					191.03 (1.297e6)
					353.18 (1.072e6)
4	Eugenol	5.41	C10H12O2	164.20	106.97 (1.205e6)
					118.97 (1.580e6)
					135.00 (2.148e6)
					148.91 (2.710e6)
					163.03 (1.191e6)
5	Ferulic acid	12.90	C_10_H_10_O_4_	194.18	134.96 (9.094e5)
					149.02 (1.772e6)
					178.99 (2.815e6)
					194.99 (9.415e5)
6	Kaempferol	8.73	C_15_H_10_O_6_	286.23	106.97 (1.205e6)
					144.00 (1.679e6)
					159.00 (1.998e6)
					161.02 (1.941e6)
					194.99 (9.415e5)
					235.06 (9.530e5)
					256.97 (1.251e6)
					286.02 (4.068e6)
7.	Luteolin	7.04	C_18_H_16_O_8_	286.25	118.97 (1.580e6)
					135.00 (2.148e6)
					151.00 (9.503e5)
					161.02 (1.941e6)
					197.00 (1.807e6)
					217.08 (8.963e5)
					287.02 (4.068e6)
8.	Quercetin	10.37	C_15_H_10_O_7_	302.00	106.97 (1.205e6)
					121.97 (1.872e6)
					151.00 (9.503e5)
					163.03 (1.191e6)
					179.99 (9.772e5)
					227.02 (1.137e6)
					275.10 (1.186e6)
					299.03 (1.021e6)
9.	Rosmarinic acid	8.72	C_18_H_16_O_8_	360.08	135.00 (2.148e6)
					161.02 (1.941e6)
					179.99 (9.772e5)
					197.00 (1.807e6)
					353.18 (1.072e6)
10.	Rutin	9.72	C_27_H_30_O_16_	611.49	286.95 (2.071e6)
					299.03 (1.021e6)
					611.46 (1.373e6)
11.	Sinapic acid	4.87	C_11_H_12_O_5_	224.06	121.97 (1.872e6)
					134.96 (9.094e5)
					149.02 (1.792e6)
					164.97 (1.945e6)
					179.99 (9.772e5)
					193.00 (8.944e5)
					207.01 (9.698e5)
					227.08 (1.137e6)
12.	Ursolic acid	12.90	C_36_H_60_O_6_	456.35	375.09 (9.336e5)
					409.31 (3.133e6)
					410.31 (1.034e6)
					431.17 (2.492e6)
13.	Vitexin	10.37	C_21_H_20_O_10_	432.00	121.97 (1.872e6)
					256.97 (1.251e6)
					299.03 (1.021e6)
					313.18 (3.685e6)
					431.17 (2.492e6)

RT: retention time. (M + H)^+^: molecular ion detection on positive mode. (*m*/*z*): mass/charge.

### Target enzyme retrieval, validation, active site, and ADME prediction

We have targeted filarial GST, Trx, GR, and SOD proteins for molecular docking and simulation studies with bioactive compounds from OS leaf extract. The molecular structural properties of GST and Trx proteins were obtained from the PDBsum server. Target enzyme validation of GR and SOD was done by RAMPAGE and PDBsum PROCHECK server which confirmed that more than 99.3% of amino acids residues were in the allowed region (Table 2). Further ERRAT, ProSA, and ResProx server were used to determine the quality of 3D models. The Hydrogen bond statistics of GST, Trx, GR, and SOD had coinciding observed and expected values in VADAR analysis (Table 2). The top three binding sites of antioxidant proteins/enzymes were predicted by Metapocket 2.0 server. Before docking the drug likeness properties of the OS bioactive compounds were predicted by the Lipinski filter (Table 3) and admetSAR software. The 13 OS bioactive compounds are effectively absorbed in the gastrointestinal tract and are non-carcinogenic. The LD_50_ of OS bioactive compounds was in the range of 1.4041–3.0825 mol/kg and the toxicity (oral) values were below grade III except for vitexin and caffeic acid, thus confirming that the above mentioned OS compounds can be considered for further studies (Table 4).

**Table 2.** Analysis of targeted filarial antioxidant proteins/enzymes models.

Ramachandran plot analysis							
	Procheck server				RAMPAGE server		
Target protein	Most favoured regions	Additional allowed regions	Generously allowed regions	Disallowed regions	Number of residues in favoured region	Number of residues in allowed region	Number of residues in outlier region
GST	169 (92.3%)	13 (7.1%)	1 (0.5%)	0 (0.0%)	201 (98.0%)	3 (1.5%)	1 (0.5%)
Trx	118 (92.9%)	9 (7.1%)	0 (0.0%)	0 (0.0%)	137 (96.5%)	5 (3.5%)	0 (0.0%)
GR	352 (90.5%)	34 (8.7%)	2 (0.5%)	1 (0.3%)	431 (96.4%)	13 (2.9%)	3 (0.7%)
SOD	189 (94.5%)	10 (5.0%)	0 (0.0%)	1 (0.5%)	216 (97.7%)	4 (1.8%)	1 (0.5%)

Hydrogen bond statistics						
Target protein	Mean H-bond distance		Mean H-bond energy		Residue with H- bond	
	Observed	Expected	Observed	Expected	Observed	Expected
GST	2.2 (sd = 0.4)	2.2 (sd = 0.4)	−1.7 (sd = 1.0)	−2.0 (sd = 0.8)	175 (84%)	155 (75%)
Trx	2.2 (sd = 0.4)	2.2 (sd = 0.4)	−1.8 (sd = 1.0)	−2.0 (sd = 0.8)	121 (84%)	108 (75%)
GR	2.2 (sd = 0.3)	2.2 (sd = 0.4)	−1.9 (sd = 0.9)	−2.0 (sd = 0.8)	348 (77%)	336 (75%)
SOD	2.2 (sd = 0.3)	2.2 (sd = 0.4)	−1.7 (sd = 1.0)	−2.0 (sd = 0.8)	182 (81%)	167 (75%)

**Table 3.** Drug likeness properties of OS bioactive compounds.

Drug name	PubChem CID	Molecular weight (g/mol)	LogP value	H-bond donor	H-bond acceptor	Molar refractivity
Apigenin	5280443	270.24	2.419	3	5	70.813
Caffeic acid	689043	180.16	1.195	3	4	46.441
Chlorogenic acid	1794427	354.31	−0.645	6	9	82.518
Eugenol	3314	164.20	2.129	1	2	48.559
Ferulic acid	445858	194.00	1.498	2	4	51.328
Kaempferol	5280863	286.24	2.305	4	6	72.385
Luteolin	5280445	286.24	2.125	4	6	72.478
Quercetin	5280343	302.00	2.010	5	7	74.050
Rosmarinic acid	5281792	360.31	1.761	5	8	89.796
Rutin	5280805	610.00	−1.878	10	16	137.495
Sinapinic acid	637775	224.21	1.507	2	5	57.880
Ursolic acid	64945	456.70	7.089	2	3	132.611
Vitexin	5280441	432.40	−0.065	7	10	103.534

**Table 4.** ADMET properties of selected *Ocimum sanctum* bioactive compounds used in the docking analysis.

Parameters	Name of drug/agent												
Absorption	Apigenin	Caffeic acid	Chlorogenic acid	Eugenol	Ferulic acid	Kaempferol	Luteolin	Quercetin	Rosmarinic Acid	Rutin	Sinapinic acid	Ursolic acid	Vitexin
Blood–brain barrier	BBB+	BBB–	BBB+	BBB+	BBB–	BBB+	BBB–	BBB–	BBB+	BBB–	BBB+	BBB+	BBB–
Human Intestinal Absorption	HIA+	HIA+	HIA+	HIA+	HIA+	HIA+	HIA+	HIA+	HIA+	HIA+	HIA+	HIA+	HIA+
Caco-2 Permeability	Caco2+	Caco2+	Caco2–	Caco2+	Caco2+	Caco2–	Caco2–	Caco2–	Caco2–	Caco2–	Caco2+	Caco2+	Caco2–
P-glycoprotein Substrate	Non-substrate	Non-substrate	Substrate	Non-substrate	Non-substrate	Substrate	Substrate	Substrate	Substrate	Non-substrate	Non-substrate	Substrate	Substrate
hERG	Weak Inhibitor	Weak Inhibitor	Weak Inhibitor	Weak Inhibitor	Weak Inhibitor	Weak Inhibitor	Weak Inhibitor	Weak Inhibitor	Weak Inhibitor	Weak Inhibitor	Weak Inhibitor	Weak Inhibitor	Weak Inhibitor
AMES Toxicity	Non-AMES Toxic	Non-AMES Toxic	Non-AMES Toxic	Non-AMES Toxic	Non-AMES Toxic	Non-AMES Toxic	Non-AMES Toxic	Non-AMES Toxic	Non-AMES Toxic	Non-AMES Toxic	Non-AMES Toxic	Non-AMES Toxic	AMES Toxic
Carcinogens	Non-Carcinogens	Non-Carcinogens	Non-Carcinogens	Non-Carcinogens	Non-Carcinogens	Non-Carcinogens	Non-Carcinogens	Non-Carcinogens	Non-Carcinogens	Non-Carcinogens	Non-Carcinogens	Non-Carcinogens	Non-Carcinogens
Acute Oral Toxicity	III	IV	III	III	IV	II	II	II	III	III	III	III	IV
Rat Acute Toxicity	1.9616 LD50, mol/Kg	1.4041 LD50, mol/Kg	2.5685 LD50, mol/Kg	1.9616 LD50, mol/Kg	1.4314 LD50, mol/Kg	3.0825 LD50, mol/Kg	3.0200 LD50, mol/Kg	3.0200 LD50, mol/Kg	2.4984 LD50, mol/Kg	2.2996 LD50, mol/Kg	2.2035 LD50, mol/Kg	2.3902 LD50, mol/Kg	2.3664 LD50, mol/Kg

### Molecular docking studies

Molecular docking was performed to obtain the ideal conformation of all 13 OS bioactive compounds with GST, Trx, GR, and SOD proteins of filarial worms. The binding affinity, GSC score, AI area, and dissociation constant of the docked complexes are listed in Table 5 and the 3D visualization of the docked complexes is shown in Figure 5. The OS bioactive compound Ursolic acid had the highest binding affinity with all four filarial antioxidant proteins whereas the anti-filarial drug DEC showed the lowest binding energy. The docking studies also revealed the formation of ample hydrogen bonding of filarial targets with OS bioactive compounds. The interacting amino acid residues were mostly present in the predicted binding sites of antioxidant enzymes and proteins (Figure 5). The computed binding energy of ursolic acid was the highest among all the ligands and anti-filarial drugs. The binding energy of ursolic acid was 7.8, 8.0, 9.4 and 7.6 kcal/mol with GST, Trx, GR and SOD, respectively.

Figure 5.Visualization of 3D interaction of filarial antioxidant enzyme/proteins with bioactive compounds of *Ocimum sanctum.* A: GST; B: Trx; C: GR; D: SOD.

**Figure:**
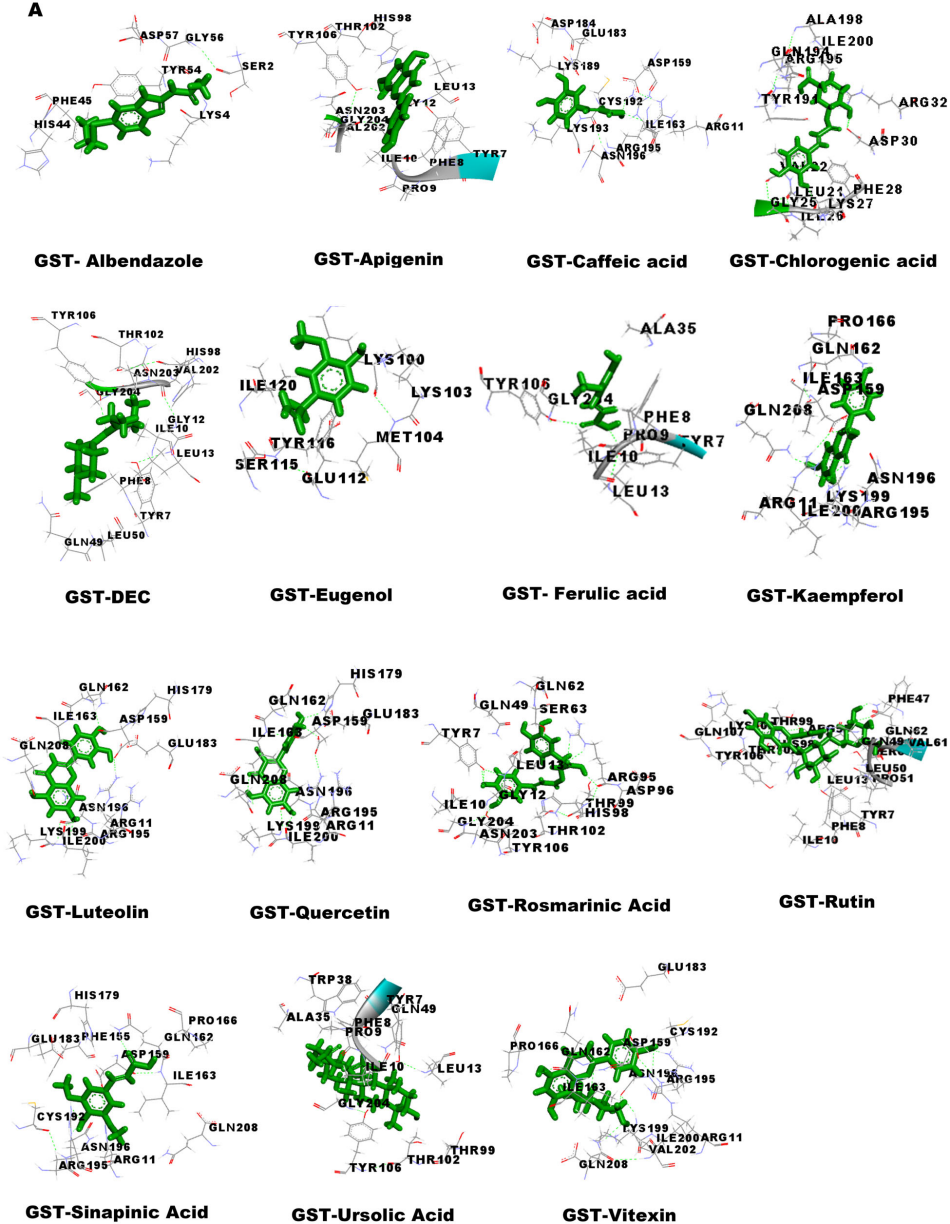

**Figure:**
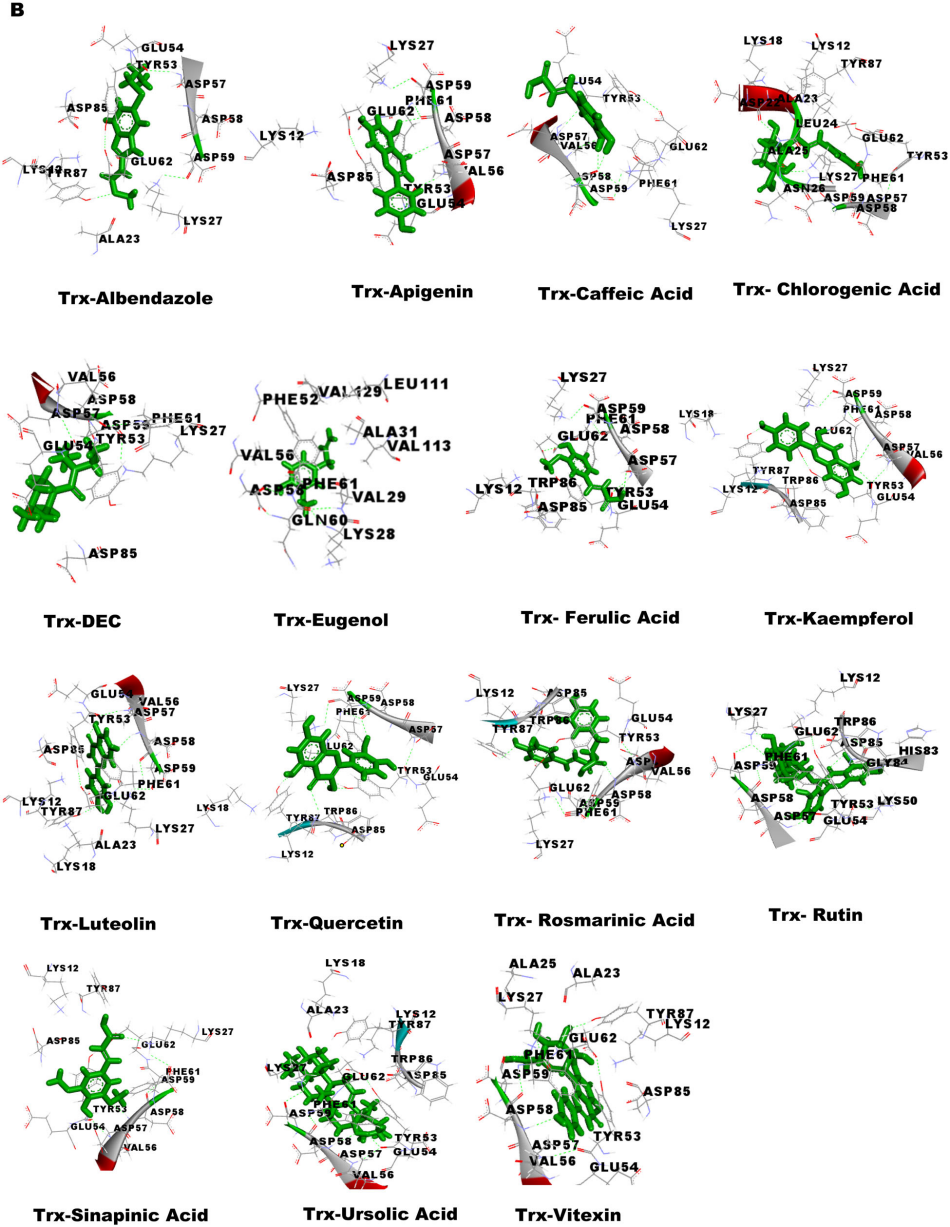

**Figure:**
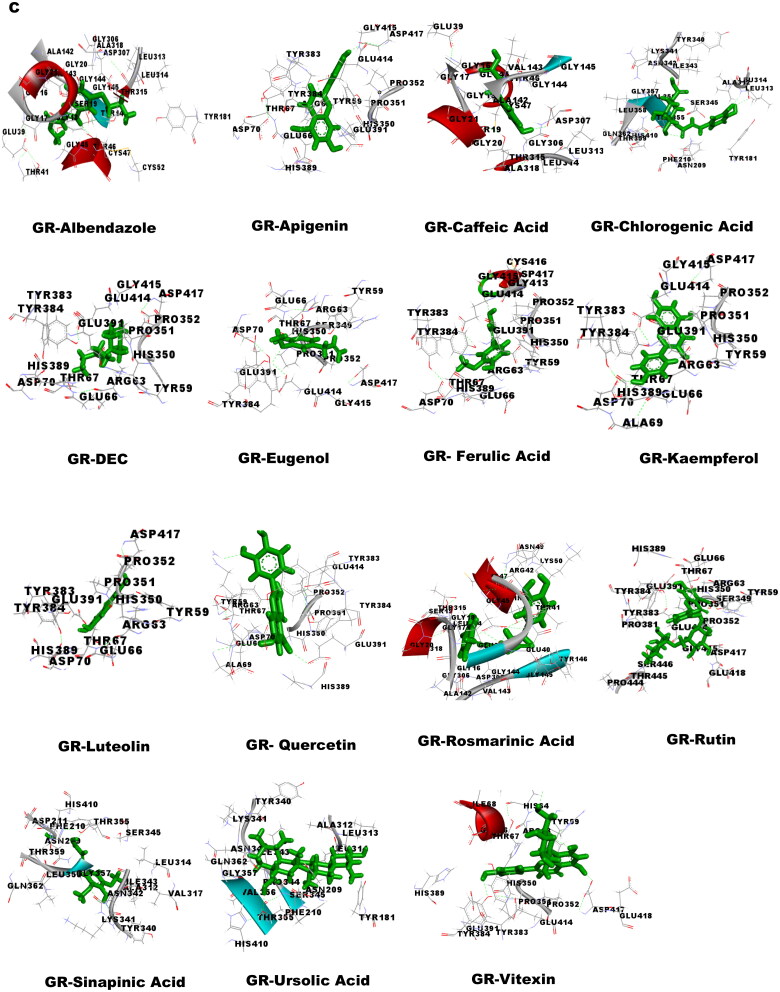

**Figure:**
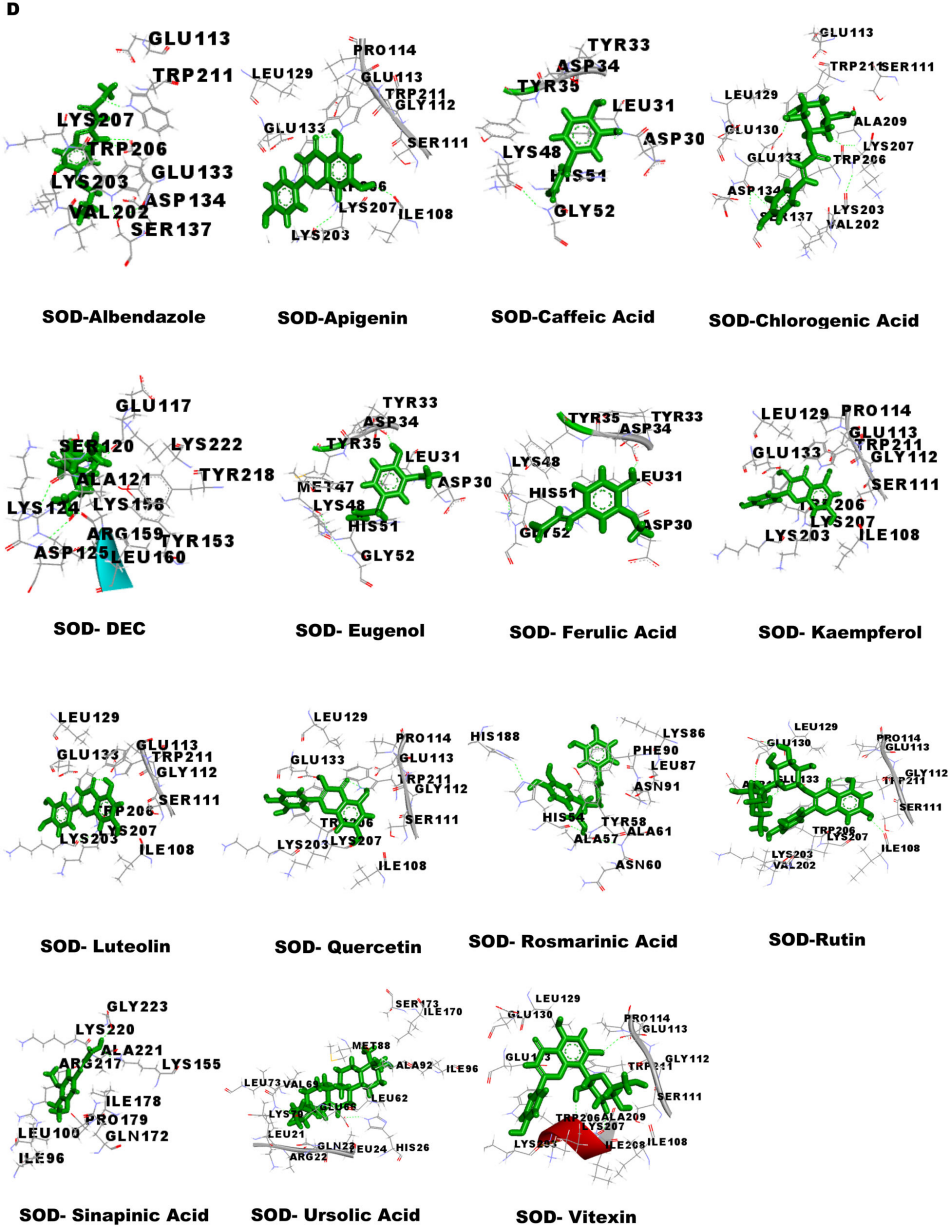

**Table 5.** Docking summary of GST, TRX, GR and SOD with bioactive compounds of *Ocimum sanctum.*

Receptor	Name of ligand	Binding energy (Kcal/mol)	Dissociation constant (um)	GSC score	AI area	Receptor	Name of ligand	Binding energy (Kcal/mol)	Dissociation constant (um)	GSC score	AI srea
GST	Albendazole	5.2910	132.3261	3540	466.60	Trx	Albendazole	5.4880	94.8948	3374	363.30
	Apigenin	6.9460	8.1005	3562	476.90		Apigenin	6.8120	10.1563	3262	366.90
	Caffeic acid	5.5750	81.9352	2950	364.60		Caffeic acid	5.8380	52.5641	2536	261.40
	Chlorogenic acid	6.7450	11.3723	4058	501.30		Chlorogenic acid	7.1000	6.2464	3510	398.50
	DEC	4.2150	813.5121	3314	414.10		DEC	4.5210	485.3589	3000	320.20
	Eugenol	4.8010	302.566	3240	404.60		Eugenol	5.5730	82.212	2816	344.30
	Ferulic acid	5.4180	106.4955	3222	375.70		Ferulic acid	5.7270	63.3947	2830	301.90
	Kaempferol	6.7270	11.7231	3616	411.00		Kaempferol	7.1110	6.131516	3324	370.30
	Luteolin	6.9170	8.5069	3430	431.00		Luteolin	7.0590	6.693979	3466	373.00
	Quercetin	6.9470	8.0868	3572	418.20		Quercetin	7.2540	4.8166	3238	340.90
	Rosmarinic acid	6.8560	9.4294	4602	580.80		Rosmarinic acid	7.1820	5.4390	3896	456.40
	Rutin	5.4810	96.0226	2946	368.80		Rutin	5.7340	62.6501	2582	280.10
	Sinapic acid	5.6590	71.1046	3286	438.90		Sinapic acid	5.6490	72.3149	2948	371.10
	Ursolic acid	7.8280	1.8280	4450	651.90		Ursolic acid	8.0640	1.2274	4398	476.80
	Vitexin	7.5580	2.8834	4226	443.80		Vitexin	7.2140	5.1530	4188	474.30
GR	Albendazole	7.5140	3.1057	4806	530.80	SOD	Albendazole	5.8040	55.6687	3648	413.90
	Apigenin	8.6520	0.4549	4512	508.40		Apigenin	6.6020	14.4767	3322	399.20
	Caffeic acid	6.6040	14.4279	3268	355.00		Caffeic acid	5.5470	85.9003	2590	291.20
	Chlorogenic acid	7.8880	1.6520	5332	602.20		Chlorogenic acid	7.3400	04.1658	3696	415.80
	DEC	5.6920	67.2525	3918	434.80		DEC	4.2520	764.2627	2936	342.30
	Eugenol	5.5030	92.5225	3578	390.80		Eugenol	4.8850	262.571	3004	354.50
	Ferulic acid	6.3520	22.0760	3530	395.10		Ferulic acid	5.4440	102.2104	2936	346.50
	Kaempferol	8.8850	0.3070	4440	499.30		Kaempferol	6.4030	20.2553	3236	389.20
	Luteolin	8.2530	0.8922	4292	489.40		Luteolin	6.8130	10.1392	3338	398.90
	Quercetin	8.2740	0.8611	4518	508.70		Quercetin	6.4650	18.2428	3232	431.40
	Rosmarinic acid	9.3240	0.1463	5452	616.20		Rosmarinic acid	6.5900	14.7729	3992	415.00
	Rutin	6.0250	38.3368	3390	368.10		Rutin	4.8950	258.1769	2892	337.80
	Sinapic acid	6.1280	32.2193	3878	452.50		Sinapic acid	4.9080	252.5738	2974	341.10
	Ursolic acid	9.4170	0.1250	5654	720.40		Ursolic acid	7.6040	02.6680	4410	492.60
	Vitexin	8.5930	0.5026	4710	529.40		Vitexin	6.7140	11.9831	3790	492.00

### Molecular dynamics simulation analysis

**Figure 6.**
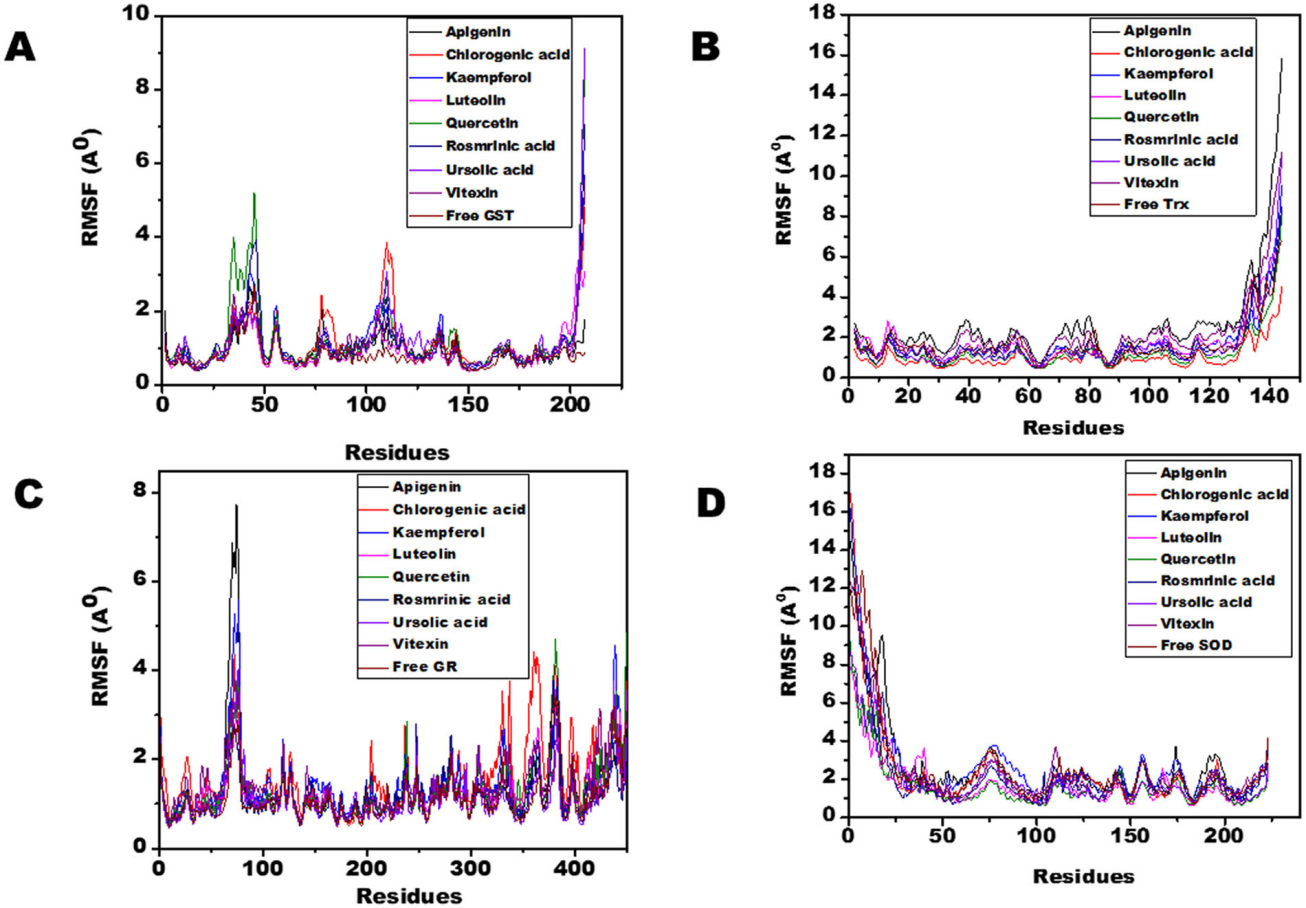
The RMSF of filarial antioxidant proteins/enzymes with OS bioactive compounds as a function of time (50 ns). (A) RMSF analysis of amino acid residues of complexed and free GST. (B) RMSF analysis of amino acid residues of complexed and free Trx. (C) RMSF analysis of amino acid residues of complexed and free GR. (D) RMSF analysis of amino acid residues of complexed and free SOD.

**Figure 7.**
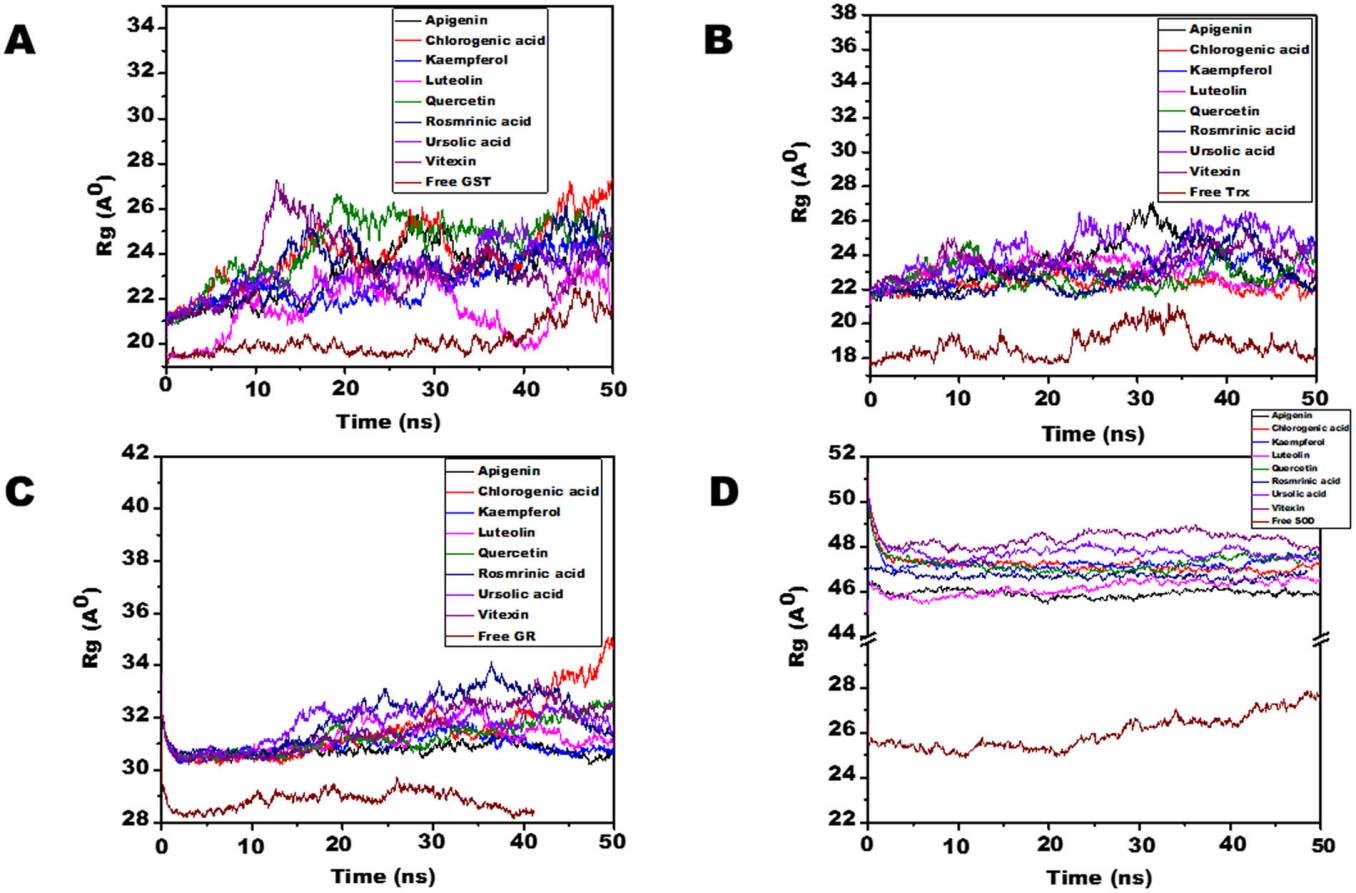
The radius of gyration (Rg) of filarial antioxidant proteins/enzymes with OS bioactive compounds as a function of time (50 ns). (A) Rg of complexed and free GST. (B) Rg of complex and free Trx. (C) Rg of complex and free GR. (D) Rg of complex and free SOD.

**Figure 8.**
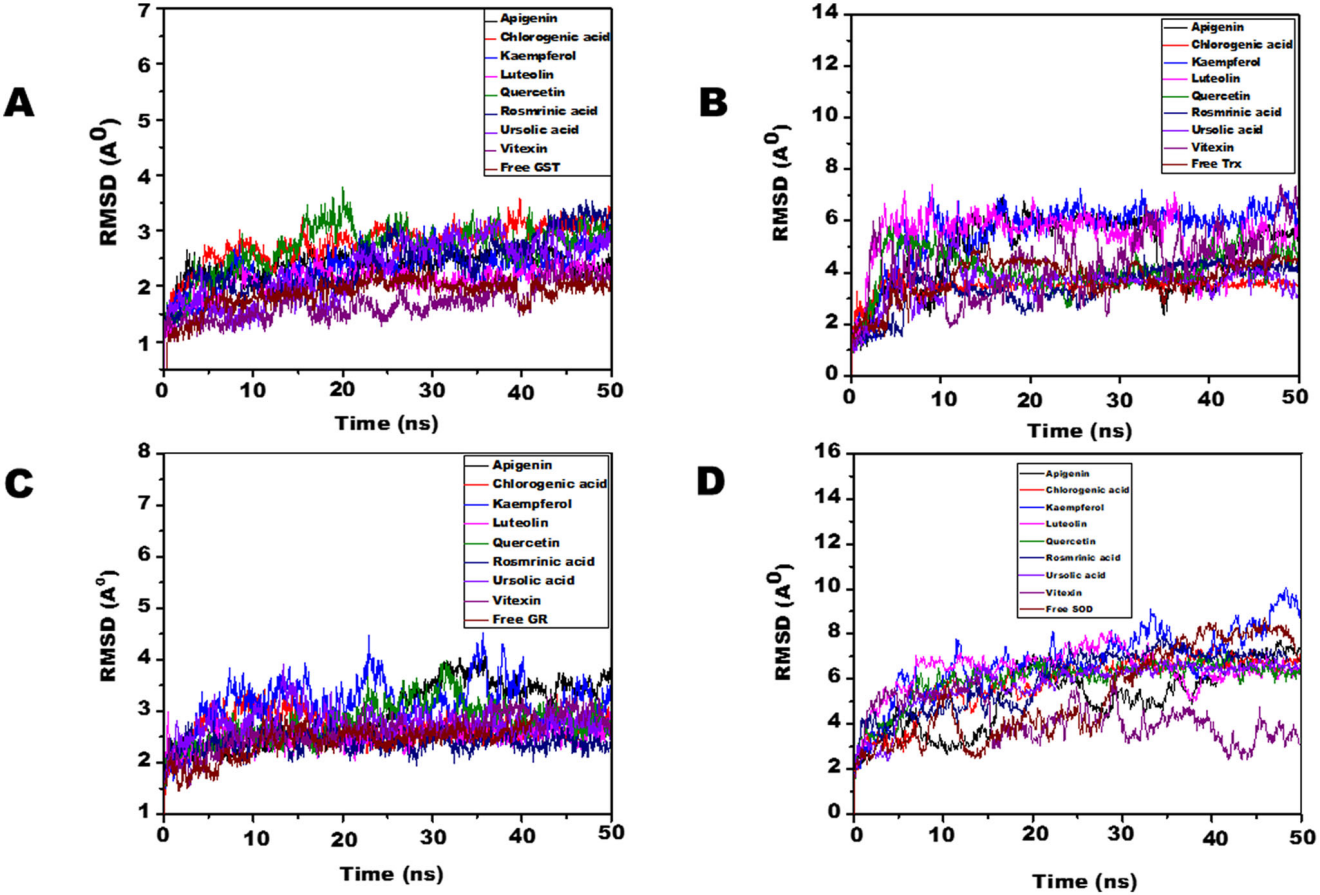
The RMSD of filarial antioxidant proteins/enzymes complexed with OS bioactive compounds as a function of time (50 ns). (A) RMSD of Cα atoms of complexed and free GST. (B) RMSD of Cα atoms of complexed and free Trx. (C) RMSD of Cα atoms of complexed and free GR. (D) RMSD of Cα atoms of complexed and free SOD.

Molecular dynamic simulations of only 8 OS bioactive compounds, apigenin, chlorogenic acid, kaempferol, luteolin, quercetin, rosmarinic acid, ursolic acid, and vitexin was performed. These compounds were chosen for MD run based on higher binding energy and GSC scores in molecular docking. The MD simulations of individual filarial GST, Trx, GR and SOD with water were utilized as a control for respective targets. The MD run was carried out for 50 nanoseconds (ns) in an isothermal-isobaric (NPT) ensemble (310 K and 1 bar). The RMSF graphs of GST, Trx, GR and SOD are represented in Figure 6. The RMSF of GST with OS bioactive compounds chosen for MD simulation was stable except for the minor fluctuations shown in amino acid residues from 106 to 112. The *B. malayi* GR complexed with OS bioactive compounds showed slight fluctuations of amino acid residues from 65 to 80. The RMSF of SOD bound complexes fluctuated between 2 and 18 amino acid residues initially but later lesser fluctuations were observed over the entire run. The Trx complexed with OS bioactive compounds exhibited least RMSF out of all the four antioxidant proteins. The RMSF per residue calculated for the liganded complexes of GST, Trx, GR, and SOD was comparable to the unliganded proteins thus conforming no major perturbations in the protein structures after binding of *Ocimum* bioactive compounds. The radius of gyration (Rg) representing the compactness of the complexed filarial antioxidant proteins with OS bioactive compounds during MD simulation is given in Figure 7. The average radius of gyration of Luteolin with GST was 21.8 Å which was the least among all bioactive compounds. Apigenin complex with GR and SOD had the most compact structure with an average Rg value of 26.3 Å and 22.9 Å, respectively. Furthermore, Trx complexed with chlorogenic, acid had the least Rg value of 22.4 Å whereas the maximum Rg 24.2 Å was given by Trx complexed apigenin. The radius of gyration plots shows an increase in Rg of the OS bioactive compounds complexed with filarial proteins/enzymes. The root mean square deviations of the docked filarial proteins/enzymes with OS bioactive compounds are detailed in Figure 8. The overall total energy, potential energy volume, and temperature were stable in the entire MD simulation run of 50 ns. The RMSD values of the docked complexes of anti-filarial proteins/enzymes with OS bioactive compounds were always within a range of 10 Å. The RMSD of GST was in the range of 1.8–3.4 Å for the entire MD simulation run. The interaction of Trx with chlorogenic acid was stabilized at RMSD of 3.6 Å while with other OS bioactive compounds the range was 3.8–7.5 Å. The RMSD values for GR with apigenin, chlorogenic acid, kaempferol, luteolin, quercetin, rosmarinic acid, ursolic acid, and vitexin varied from 2 Å to 4.4 Å. It was seen that the Apigenin–SOD complex was most stable with an average RMSD value of 5 Å followed by other OS compounds.

## Discussion

LF infection results in severe pathologies like lymphedema, hydrocele, lymphangitis, elephantiasis, and tropical pulmonary eosinophilia. The WHO recommends IDA program (ivermectin, DEC and albendazole) for LF elimination and control. These drugs are only effective against microfilariae and have negligible effect on adult worms. The IDA treatment is often associated with various side effects like transient fever, headache, dizziness, malaise, myalgia, fatigue, and gastrointestinal problems (Budge et al. 2018). Less common side effects are cough, dyspnoea, blood-tinged sputum, bronchoconstriction, urticaria, haematuria, etc. DEC is reported to cause loss of vision and fatal encephalopathy when given to the patients co-infected with onchocerciasis or loiasis (Fischer et al. 2017; Gobbi et al. 2019). Several reports describe ivermectin related serious adverse drug reactions (SADRs) including liver and kidney dysfunction (Ampillo et al. 2021). Therefore, anti-filarial drugs that are effective against adult filarial worms are urgently needed for LF treatment.

Previously, *Ocimum sanctum* (OS) plant has been shown to have anthelminthic effects against gastrointestinal nematodes of sheep and ruminants (Kanojiya et al. 2015; Sousa et al. 2021). In another study, Eugenol and other essential oils of OS have shown anthelminthic activity against the free-living nematode *Caenorhabditis elegans* (Asha et al. 2001). *Ocimum sanctum* (OS) is widely known since ancient times for its medicinal properties. OS is a diverse medicinal plant having antioxidant, anti-inflammatory, immunomodulatory, antidiabetic, and antimicrobial properties (Pattanayak et al. 2010). In the present work, we have investigated the anti-filarial activity of OS and its bioactive components against filarial parasites. The *in vitro* studies were done against bovine filarial parasites which is an established model for lymphatic filariasis (Sharma et al. 2022), whereas for *in silico* studies the antioxidant enzymes/proteins were retrieved from *W. bancrofti* and *B. malayi* databases.

In the study, treatment of female *S. cervi* parasites with EOS resulted in significant motility reduction of parasites which could be due to the oxidative stress generated by *Ocimum*. It is known that excessive production of ROS can cause oxidation of cellular macromolecules in turn impairing the normal cellular functioning and culminating into cell death. Earlier, EOS was reported to induce DNA fragmentation and apoptosis in A549 lung cancer cells under *in vitro* conditions (Wihadmadyatami et al. 2019). Furthermore, *Ocimum sanctum* extract has proven effective in inducing apoptosis in lung carcinoma, colorectal adenocarcinomas and lung epithelial cells (A549). We observed that treatment of *S. cervi* with EOS resulted in DNA fragmentation and increase in ROS levels both of which are hallmarks for apoptosis and programmed cell death.

*Ocimum sanctum* is rich in phytochemicals and contains many bioactive and nutraceuticals molecules that differ from strain to strain and even differ from place to place. The variation in bioactive compounds of *Ocimum* is common to different growing locations, environmental conditions, and geographical origins. Although most researchers reported ursolic acid, eugenol and rosmarinic acid as major constituents of OS leaf extract (Pandey et al. 2015; Prinsi et al. 2019). The presence of apigenin, chlorogenic acid, eugenol, luteolin, quercetin, rosamrinic acid, and ursolic acid prove that the EOS used in this study would be having antioxidant activity (Farouk et al. 2016). All the 13 bioactive compounds identified by UPLC-ESI-MS/MS from EOS were docked against filarial antioxidant proteins/enzymes as targets.

The filarial parasites have a strong antioxidant defense system which helps in evading the host oxidative attack mechanisms. In filarial parasites, superoxide dismutases are the first line of defense against ROS produced either internally or by external sources (Rathaur et al. 2001). Superoxidase dismutases protect the parasites from superoxide anion by converting it to molecular oxygen and hydrogen peroxide. The cytochrome P-450 system is completely absent in filarial parasites and instead, GST, a GSH requiring enzyme, plays a key role in the detoxification of xenobiotics (Birben et al. 2012). Previous researches have successfully demonstrated GST as a potential drug and vaccine target (Gupta and Srivastava 2006; Rathaur et al. 2008). Another antioxidant enzyme glutathione reductase maintains a constant availability of reduced glutathione (GSH) by continuously reducing GSSG to GSH, thus maintaining an anti-oxidative environment essential for the survival of the parasites (Gupta and Srivastava 2006). Filarial parasites also have a second line of defense comprising thioredoxins like the thioredoxin reductase and thioredoxin peroxidase which are established drug targets of LF (Anand et al. 2012; Tiwari et al. 2015).

Molecular docking was performed with filarial glutathione *S*-transferase (GST), thioredoxin (Trx), glutathione reductase (GR), and superoxide dismutase (SOD) which are key antioxidant enzymes/proteins involved in the defense system of filarial worms. Molecular docking is a faster option for the discovery of novel drugs and is an inexpensive alternative to tedious *in vitro* drug screening procedures. The maximum interactions of *Ocimum* bioactive compounds with filarial antioxidant proteins were present within the active site of GST, Trx, GR, and SOD (Figure 5). It is an established fact that binding of ligands within the active site of target molecules can lead to modulations in enzymatic functions and molecular properties of proteins (Schena et al. 2015). We have also analyzed the H-bonding between OS bioactive compounds and target proteins and the maximum number of H-bonds were formed between GST and rosmarinic acid. Further, rutin, vitexin, chlorogenic acid, and quercetin formed hydrogen bonding with at least 3 of the 4 filarial antioxidant enzymes/proteins studied showing their stronger interactions as compared to other OS compounds. We analyzed the binding of OS bioactive compounds with filarial antioxidant proteins with YASARA and PatchDock software. Besides ursolic acid, which showed highest binding energy with all four targets, vitexin, chlorogenic acid, quercetin, and rosmarinic acid also had higher binding energies and larger AI areas. In fact, almost all the OS bioactive compounds except caffeic acid and eugenol showed higher binding energies and lower dissociation constants as compared to the established anti-filarial drug albendazole and DEC.

To further investigate the stability of interactions between eight OS bioactive compounds and filarial antioxidant enzymes/proteins we performed molecular dynamics simulation. The simulation data obtained in this study clearly shows that OS bioactive compounds remained bound to the proteins throughout the run suggesting that these complexes are well stabilized. Ursolic acid, chlorogenic acid, and luteolin were most stable during the simulation run showing the lowest RMSD and RMSF values. Ursolic acid (UA) has preventive and curative properties against many diseases affecting the liver and brain, and also in cancers and metabolic syndromes etc. (Seo et al. 2018). UA has been found to be effective against *B. malayi* GST in a previous study (Kalani et al. 2014). Luteolin has antioxidant properties and has shown potential as anti-inflammatory agent in a gut inflammation model (Mizuno and Nishitani 2013). Chlorogenic acid is an important bioactive polyphenol and many studies confirm it as antidiabetic, anti-obesity, anti-tumor, neuro protector, hepatoprotective, antioxidant, and anti-inflammatory activity (Tajik et al. 2017). Therefore, the combined results of these *ex vivo* and *in silico* studies with OS and its bioactive compounds strongly indicate towards their anti-filarial potential which should be explored further for developing adulticidal filarial drugs.

## Conclusions

Lately, the use of *Ocimum species* for the treatment of parasitic diseases is being explored extensively due to higher efficiency and fewer side effects as compared to allopathic drugs. In this report, the three most promising OS bioactive compounds having anti-filarial activity were identified as chlorogenic acid, luteolin, and ursolic acid. The overall findings from our study provide an understanding of the effect of OS and EOS on filarial parasites. The antioxidant properties and multi-inhibitory potential of EOS and OS bioactive compounds against filarial proteins/enzymes indicates that further studies may lead to development of better anti-filarial treatment strategies.
